# Stents and Emerging Alternatives in Upper Gastrointestinal Endoscopy: A Comprehensive Review

**DOI:** 10.3390/diagnostics15182344

**Published:** 2025-09-16

**Authors:** Francesca Bernardi, Giuseppe Dell’Anna, Paolo Biamonte, Alberto Barchi, Lorella Fanti, Alberto Malesci, Lorenzo Fuccio, Emanuele Sinagra, Giulio Calabrese, Antonio Facciorusso, Angelo Bruni, Gianfranco Donatelli, Silvio Danese, Francesco Vito Mandarino

**Affiliations:** 1 Gastroenterology and Gastrointestinal Endoscopy Division, IRCCS San Raffaele Hospital, Via Olgettina 60, 20132 Milan, Italy; bernardi.francesca@hsr.it (F.B.); dellanna.giuseppe@hsr.it (G.D.); biamonte.paolo@hsr.it (P.B.); barchi.alberto@hsr.it (A.B.);; 2 Faculty of Medicine and Surgery, Vita-Salute San Raffaele University, Via Olgettina 56, 20132 Milan, Italy; 3 Gastroenterology Unit, IRCCS Azienda Ospedaliero-Universitaria di Bologna, 40138 Bologna, Italy; 4 Gastroenterology Unit, Fondazione Istituto San Raffaele Giglio, 90015 Cefalù, Italy; 5 Gastroenterology and Endoscopy Unit, ARNAS Civico Di Cristina Benfratelli, 90127 Palermo, Italy; 6 Clinical Medicine and Surgery, University of Naples Federico II, 80138 Naples, Italy; donatelligianfranco@gmail.com; 7 Gastroenterology Unit, Faculty of Medicine and Surgery, University of Salento, 73100 Lecce, Italy; 8 Unité d’Endoscopie Interventionnelle, Ramsay Santé, Hôpital Privé des Peupliers, 75013 Paris, France

**Keywords:** SEMS, stent, endoscopy, EVT, LAMS, leak, strictures

## Abstract

Endoscopy has revolutionized the management of gastrointestinal (GI) conditions, enabling less invasive treatments for cases that once required surgery. Among these innovations, endoscopically placed stents have played a crucial role in the treatment of upper GI tract diseases for many years. Today, stents remain a valid first-line treatment for specific indications; however, advancements in endoscopic technologies have led to a reassessment of their role in some conditions. While stents are still the primary choice for palliation of malignant esophageal strictures, endoscopic vacuum therapy (EVT) has demonstrated superior outcomes for esophageal leaks, and Endoscopic UltraSonography-guided placement of lumen-apposing metal stents (LAMS) has outperformed traditional stents in gastric obstructions. This review evaluates current stent indications, highlighting upper GI conditions where they remain the best option, while also exploring emerging technologies and updated clinical guidelines to optimize patient care.

## 1. Introduction

Endoscopic stenting has become a cornerstone in the management of a broad spectrum of gastrointestinal (GI) conditions [1,2]. These devices are primarily used to restore and maintain luminal patency in cases of obstruction, perforation, leaks, or strictures throughout the upper GI tract, including the esophagus, stomach, and duodenum [1]. Advances in stent design and technology have significantly enhanced the therapeutic potential of endoscopy, allowing for the effective treatment of conditions that once required open surgery.

Over the decades, stent use has evolved, driven by ongoing innovation and growing evidence supporting their efficacy. However, the advent of advanced endoscopic techniques has prompted a re-evaluation of stents as first-line therapy in selected scenarios. Approaches such as endoscopic ultrasound (EUS)-guided procedures and vacuum-assisted therapies have gained traction for addressing the limitations of stents in cases with higher complication risks or suboptimal outcomes [3]. For instance, EUS-guided gastroenterostomy (EUS-GE) has demonstrated superior efficacy in managing malignant gastric outlet obstruction (GOO) and complex post-surgical anatomies [3]. Likewise, Endoscopic Vacuum Therapy (EVT), which applies continuous negative pressure to promote tissue healing, has proven highly effective in treating anastomotic leaks in the upper GI tract, where stents may fail or carry additional risks [4,5].

As therapeutic endoscopy continues to advance, it becomes increasingly important to define the respective roles of traditional stenting and emerging techniques. This review offers a comprehensive overview of the current landscape in 2025, analyzing stent types, indications, and clinical outcomes, while also exploring the expanding role of innovative endoscopic approaches.

## 2. Type of Stents

Endoscopic stents are primarily categorized into three types: metallic stents, plastic stents, and biodegradable stents. Each type offers specific advantages tailored to distinct clinical scenarios. Currently, metallic stents are the most widely used, while plastic stents have largely fallen out of favor.

### 2.1. Metallic Stent

Metallic stents, also known as self-expanding metal stents (SEMSs), are primarily composed of alloys such as nitinol (nickel–titanium), stainless steel, or cobalt–chromium [6]. Nitinol is especially valued for its superelasticity and shape-memory properties, which help maintain luminal patency even in anatomically challenging regions of the GI tract. Its resistance to deformation makes it ideal for dynamic areas like the esophagus and colon. While stainless steel and cobalt–chromium stents were more commonly used in the past, nitinol has largely replaced them due to its superior flexibility. However, cobalt–chromium stents, known for their strong radial force and corrosion resistance, are still occasionally used, particularly in vascular applications [6].

SEMS feature a flexible, open-mesh structure that provides luminal support while minimizing the risk of tissue embedding or overgrowth. The radial force they exert helps maintain lumen patency, even in the presence of significant obstruction [6].

SEMSs are generally classified into three types:

- Fully covered (FC-SEMS): Encapsulated in a plastic or silicone membrane to prevent tissue ingrowth, facilitating easier removal or repositioning. However, they are associated with a higher risk of migration.
- Partially covered (PC-SEMS): Designed with uncovered ends to reduce migration risk, though this increases the potential for tissue overgrowth and embedding.
- Uncovered (UC-SEMS): These integrate more firmly with the surrounding tissue, providing long-term support but carrying a significant risk of complications such as tissue ingrowth [7].

FC-SEMS and PC-SEMS are commonly used for the treatment of esophageal leaks, wall defects, and benign strictures due to their removability [8]. ESGE guidelines also recommend their use in the palliation of esophageal cancer, as they reduce the risk of tissue ingrowth. Conversely, UC-SEMSs are more often employed in the palliative treatment of malignant colonic obstructions, where long-term support is necessary [2].

In all indications, SEMSs help relieve symptoms and improve quality of life, particularly in patients who are not candidates for surgery or where surgery is not preferred [1,2,9].

### 2.2. Plastic Stent

Plastic stents, typically made from polymers such as polyethylene, polyurethane, or silicone, were historically used in GI endoscopy but are now infrequently employed due to their limitations. Unlike SEMS, plastic stents are non-expanding and rely on their preformed shape to maintain luminal patency [7].

Today, their role—particularly in esophageal applications—has diminished, as metallic and biodegradable stents have proven to be more effective and reliable alternatives.

### 2.3. Biodegradable Stents

Biodegradable stents (BDSs) represent a newer class of endoscopic devices designed to degrade naturally within the body through hydrolysis, breaking down into non-toxic byproducts such as carbon dioxide and water. This characteristic eliminates the need for removal procedures, reducing patient risk and procedural burden.

They are primarily composed of the following materials:

- Polylactic acid (PLA): Offers a controlled degradation rate, maintaining structural integrity for weeks to months, depending on clinical needs.
- Polyglycolic acid (PGA): Degrades more rapidly but is less mechanically stable than PLA; it is often combined with PLA to optimize performance.
- Polycaprolactone (PCL): Used in specific cases where extended durability is required [10].

BDS are available in both self-expanding and pre-shaped designs. They are particularly suitable for the management of benign strictures where prolonged luminal patency is not necessary, and where conventional stents pose risks of migration or complications upon removal. Emerging clinical evidence supports their safety and effectiveness, particularly in minimizing complications such as tissue ingrowth [10,11].

### 2.4. Lumen-Apposing Metal Stent (LAMS)

Lumen-apposing metal stents (LAMSs) are specialized self-expanding metal stents designed for endoscopic drainage and the creation of anastomoses between adjacent luminal structures [12]. They are primarily made of nitinol, a nickel–titanium alloy valued for its shape memory and superelastic properties [12]. This material allows the stent to expand into a predetermined shape upon deployment while maintaining flexibility and resistance to external compression [12]. Most LAMSs are fully covered with a silicone or polymer membrane to prevent tissue ingrowth and facilitate removal once no longer needed.

LAMSs are characterized by a fully covered, self-expanding structure with bilateral flared flanges, which ensure secure apposition between adjacent structures and minimize the risk of migration [12,13]. The stent lumen typically ranges from 6 mm to 20 mm in diameter, depending on the clinical indication.

Two main types of LAMSs are available, based on flange design [12,13]:

- Saddle-shaped LAMS: Featuring wide flanges that provide strong tissue apposition, these are commonly used for drainage of pancreatic fluid collections.
- Dumbbell-shaped LAMS: With a more symmetrical design, these are optimized for gastrointestinal bypass procedures.

Variations in stent length, diameter, and radial force allow for customization according to the target anatomy. LAMSs are typically deployed under endoscopic ultrasound (EUS) guidance, or under direct endoscopic visualization, using a preloaded catheter-based delivery system (usually via an 8F or 10F applicator) [12,13].

Due to their unique design, LAMSs enable efficient drainage of pancreatic pseudocysts and walled-off necrosis, facilitate direct endoscopic necrosectomy, and allow biliary or gallbladder drainage in patients unfit for surgery or with failed endoscopic retrograde cholangiopancreatography (ERCP). Furthermore, they are increasingly used in gastrointestinal bypass procedures. Emerging evidence also supports their off-label use in the treatment of benign GI strictures.

## 3. Esophagus

### 3.1. Esophageal Leak

Esophageal anastomotic leaks (ALs) are defined as “a full-thickness wall defect involving the esophagus, anastomosis, suture line, or conduit”. They are classified into severity grades:

- Type I: Localized leaks manageable with medical therapy;
- Type II: Requiring radiological or endoscopic intervention;
- Type III: Necessitating surgical intervention [14].

Post-esophagectomy leaks occur in approximately 10% of patients [15]. Management strategies depend on the timing of diagnosis, clinical status, and presence of necrosis or conduit ischemia. In stable, non-septic patients, endoscopic treatment is preferred over surgery due to the high morbidity of reoperations [1].

#### 3.1.1. Role of Stent

##### Indications and Mechanism of Action

According to ESGE guidelines, temporary SEMS placement may be considered for esophageal leaks, fistulas, or perforations larger than 20 mm, though no specific type is universally recommended, and stent duration should be tailored to the clinical scenario [1]. SEMSs are also indicated for sealing malignant tracheoesophageal or bronchoesophageal fistulas [1].

**Figure 1:**
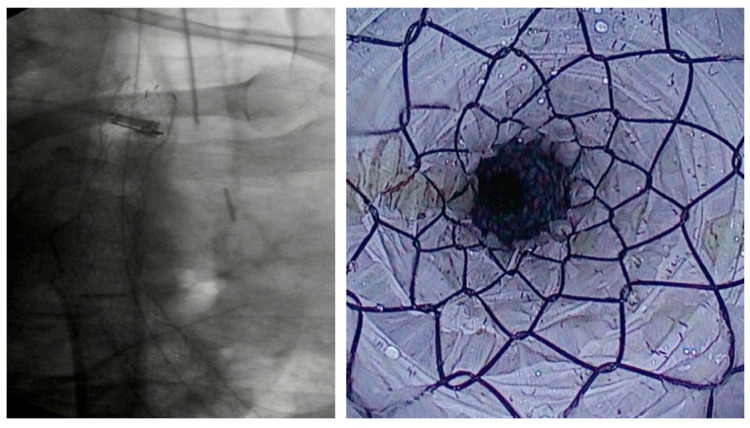
Placement of an FC-SEMS in a patient with esophagojejunal anastomotic fistula following gastrectomy.

Over the past two decades, SEMSs have become the most widely used endoscopic therapy for esophageal wall defects [16,17]. They promote healing by creating a mechanical barrier against secretions and by enabling early refeeding—critical in preventing malnutrition, which negatively impacts outcomes [18]. SEMSs typically reach full expansion within 24–48 h and are usually maintained in place for 6–8 weeks [8] [Figure 1]. The most commonly used types are FC-SEMS and PC-SEMS.

##### Efficacy and Adverse Events

FC-SEMS and PC-SEMS demonstrate clinical success (CS) rates exceeding 60% in achieving closure of esophageal fistulas and leaks [16].

In a study by Anderloni et al., involving 49 patients with ALs following esophageal surgery treated with SEMS, the CS rate was 60.5%, with no significant differences between FC-SEMS and PC-SEMS (57.1% vs. 64.7%, respectively). The overall adverse events (AEs) rate was 38.8% [19].

Similarly, Plum et al. reported a 70% CS rate in 70 patients with ALs following Ivor Lewis esophagectomy treated with FC-SEMS. The median stent indwelling time was 28 days (range 7–87), and AEs occurred in 28.6% of cases [20].

In another study by Segura et al., involving 23 patients treated for ALs or esophageal perforations, the CS rate reached 75% [21].

Despite these favorable outcomes, SEMS placement is associated with several complications. Stent migration remains the most frequent, occurring in 10% to 40% of cases, with significantly higher rates—up to 62%—in cervical anastomoses due to anatomical and functional challenges [21,22,23]. Migration is often related to a mismatch between the stent and esophageal lumen diameter [24].

To mitigate this, larger-caliber or innovatively designed FC-SEMSs have been developed. For instance, the Niti-S Beta Esophageal Stent (Taewoong Medical, Gimpo, Republic of Korea), with its dual-layer coating and increased diameter, aims to reduce migration. At our institution, a study involving 37 patients with post-esophagectomy ALs treated with 75 double-layer FC-SEMSs (mean 2.0 ± 1.3 per patient) showed a CS rate of 62.2%, with stent migration in 22.7% of cases [17].

Other strategies to minimize stent migration include endoscopic fixation techniques, such as suturing or the use of Through-The-Scope Clips (TTSC) and Over-The-Scope Clips (OTSC).

Endoscopic suturing offers a durable and secure fixation by anchoring the stent directly to the esophageal wall. This is typically achieved using dedicated systems such as the OverStitch™ Endoscopic Suturing System (BostonScientific, Marlborough, MA, USA), which is mounted on the distal tip of a standard endoscope and allows precise suture placement under direct visualization [25]. In a study by Fujii et al., the use of OverStitch resulted in 100% technical success and a significant reduction in the migration rate—33% compared to 74% in the group treated without suturing [26]. However, this technique requires advanced endoscopic expertise and specialized equipment, which may limit its availability in routine practice [25].

TTSCs provide a simpler alternative, functioning by securing the edges of the stent directly to the surrounding mucosa [27]. They are compatible with most endoscopic platforms and are especially useful for anchoring smaller stents or reinforcing fixation in selected cases. Nonetheless, their grasping force and ability to secure large or deeply seated SEMS are limited compared to other approaches [27].

OTSCs offer a more robust anchoring solution. These clips are mounted externally on the distal end of the endoscope and are deployed after retraction of the stent or target tissue into a preloaded applicator cap. Due to their spring-loaded mechanism, OTSCs provide strong tissue capture and firm stent fixation, significantly reducing the risk of migration [28].

A meta-analysis by Papaefthymiou et al., which included 10 studies and 1014 patients, confirmed the superiority of fixation techniques over non-fixation approaches. The pooled analysis showed that fixation significantly reduced migration rates (OR 0.20, 95% CI 0.11–0.37; I^2^ = 59%, *p* = 0.01). Among the methods analyzed, suturing, OTSC, and TTSC demonstrated efficacy in lowering migration risk, with ORs of 0.23, 0.31, and 0.10, respectively [29].

Another relevant complication of SEMS placement is tissue overgrowth, which occurs in approximately 15% to 20% of cases, particularly when the stent remains in place for more than one month [30]. Overgrowth can complicate stent removal and may result in mucosal damage. Mucosal erosion and bleeding, although less frequent, are reported in about 4.3% of patients and are more commonly observed in those undergoing neoadjuvant chemoradiotherapy, where the incidence may rise to 22.5% [31,32].

Stent-related leakage represents another significant concern. The reported incidence varies across studies, ranging from approximately 10% to 40% of patients treated with SEMS for anastomotic defects [22,33,34,35]. To better classify and manage these leaks, Stephens et al. proposed a classification system in 2014 based on radiographic findings and intraoperative evaluations, identifying five distinct types of stent leaks [36]:

- Type 1: Proximal leaks due to an inadequate seal, often resolved by upsizing the stent or adding an additional one.
- Type 2: Distal retrograde leaks, commonly managed with decompression PEG, additional stenting, or a larger stent.
- Type 3: Leaks through breaches within the stent lining, typically caused by technical difficulties or suction-related trauma during placement, requiring stent replacement.
- Type 4: Leaks between adjacent stents, addressed by using a larger proximal stent.
- Type 5: Migration-related leaks, usually seen in cervical or mid-esophageal stenting without fixation, necessitating a larger stent or anchoring for stability.

This classification provides a practical framework for identifying and managing stent-related leaks [36].

Recently, the use of SEMS for the treatment of esophageal leaks and fistulas has been increasingly complemented by novel endoscopic techniques, some of which have demonstrated even greater efficacy in promoting esophageal wall healing. Among these, EVT and the VAC-stent have emerged as particularly promising options.

#### 3.1.2. Endoscopic Vacuum Therapy (EVT)

EVT was first introduced approximately 15 years ago for the treatment of colonic wall defects and is now widely applied in managing upper GI tract leaks and perforations [5]. It involves the placement of a polyurethane sponge adjacent to the defect, connected to a suction tube linked to a vacuum device generating continuous negative pressure (typically around −125 mmHg, adjustable case-by-case) [4,37]. This mechanism promotes continuous drainage, tissue revascularization, and granulation tissue formation [4,37]. The most used system is the commercially available Eso-Sponge^®^ (Boston Scientific).

**Figure 2:**
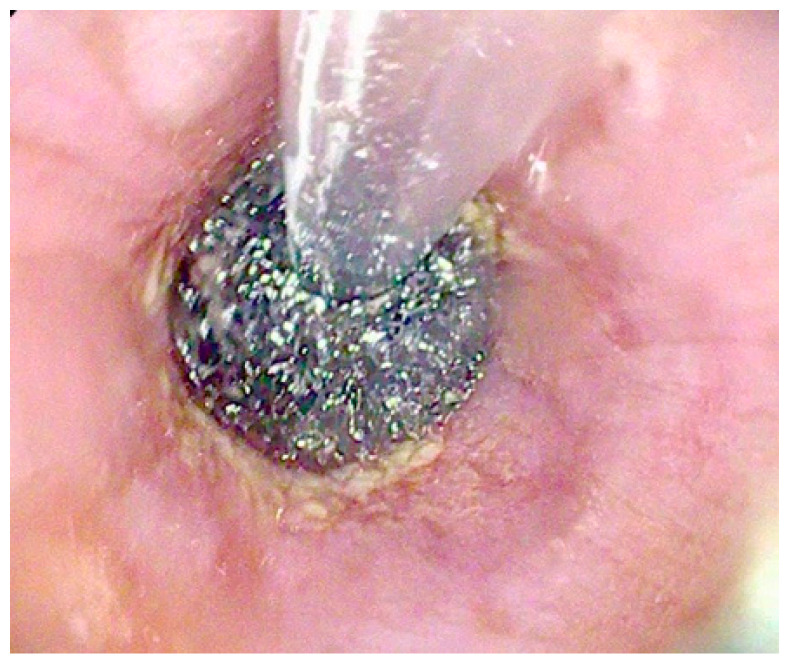
Intraluminal EsoSponge for the treatment of an anastomotic leak following Ivor Lewis esophagectomy.

The total duration of EVT is guided by periodic endoscopic reassessments, typically performed every 3–5 days during sponge replacement, and varies according to clinical response. One of EVT’s key strengths is its versatility: the sponge can be placed intraluminally in cases of small defects without collections, or intracavitary in larger defects, allowing progressive adaptation to the shrinking cavity volume [Figure 2] [4,37].

EVT has demonstrated high efficacy in the treatment of ALs and upper GI wall defects, with CS rates ranging from 90% to 100% [38,39,40].

In the study by Richter et al., including 69 patients with esophageal AL and 33 with perforations, closure rates of 91% and 76% were achieved, respectively. Mediastinitis (*p* = 0.002) and cervical location (*p* = 0.008) were significant predictors of treatment failure [41].

Similarly, in a multicenter retrospective study by Jung et al. involving 119 patients—92.4% of whom had ALs post-esophagectomy—EVT achieved a CS rate of 70.6%. The mean number of sponge replacements was 3.93 in the success group and 4.20 in the failure group. Neoadjuvant therapy and the use of intraluminal placement emerged as independent predictors of failure [38].

Multicenter series by Momblan et al. and Luttikhold et al., including 102 and 27 patients, respectively, also reported high success rates of 82% and 89% [39,40].

EVT has proven effective even as rescue therapy after failed surgical revision or endoscopic attempts. At our center, in a cohort of 12 patients with AL following unsuccessful redo surgery or prior endoscopic treatments, EVT achieved complete defect closure in 75% of cases [42].

The rate of AEs associated with EVT is generally low [43]. Jung et al. observed an AE rate of 10.9%, with sponge dislocation being the most frequent complication. Luttikhold et al. reported a 7% AE rate, including one case of iatrogenic defect enlargement during sponge exchange and one instance of bleeding requiring transfusion [40].

The most commonly reported AEs include device dislocation, mild bleeding during sponge removal, and aspiration pneumonia [38,44]. Although rare, life-threatening events such as fatal hemorrhage have been described, particularly when the sponge is positioned near major vessels, leading to erosion [45,46,47]. While only five such cases have been reported to date, they highlight a critical safety concern. In this context, thorough pre-procedural imaging to assess the anatomical relationship between the defect and nearby vascular structures is essential to reduce the risk of catastrophic events. Bronchoesophageal fistulas have also been documented, although it remains unclear whether they result from EVT itself or from the underlying ALs [45,46,47].

The most frequent long-term AE is the development of strictures at the treatment site, with an incidence ranging from 8% to 20%. These are generally manageable with endoscopic dilation [45,46,47] [Figure 2].

##### SEMS vs. EVT

Recent evidence suggests that EVT may offer superior efficacy and safety compared to SEMS in the management of post-esophagectomy ALs.

A meta-analysis by Mandarino et al., including eight comparative studies, found that EVT was associated with a significantly higher CS rate (OR 2.58, 95% CI 1.43–4.66), fewer device exchanges (pooled mean difference −4.90, 95% CI −6.71 to −3.08), shorter treatment duration (−9.18 days, 95% CI −17.05 to −1.32), and lower rates of complications (OR 0.35, 95% CI 0.18–0.71) and mortality (OR 0.47, 95% CI 0.24–0.92). However, in the subgroup analysis restricted to patients with AL after oncologic surgery, the difference in CS rates was not statistically significant (OR 1.59, 95% CI 0.74–3.40; I^2^ = 0%) [44].

A separate matched analysis by the same authors compared EVT and SEMS in the treatment of ALs < 30 mm after Ivor Lewis esophagectomy in 44 patients (22 in each group). Both techniques showed comparable CS (90.9% for EVT vs. 72.7% for SEMS), with no significant difference in the number of procedures or treatment duration. The most common complication in the SEMS group was stent migration [48].

Despite its clinical advantages, EVT has been associated with significantly higher costs compared to stent placement. In a study by Baltin et al. involving 60 patients, the mean cost per case for EVT was nearly double that of stenting (EUR 9282 vs. EUR 5156), highlighting important financial implications that warrant consideration when evaluating its broader adoption [49].

More recently, a prophylactic application of EVT—referred to as pre-emptive endoscopic negative pressure therapy (pENP)—has emerged as a promising strategy to prevent ALs in high-risk patients undergoing esophageal surgery [50]. In a study by Müller et al., 73% of 67 patients treated with pENP following minimally invasive Ivor-Lewis esophagectomy had an uneventful recovery, while 19% required prolonged EVT to achieve complete healing. The overall rate of ALs was 7.5% [51].

To conclude, although EVT appears more effective than SEMS for treating ALs and is associated with fewer complications, its higher costs remain a concern. Randomized controlled trials on larger populations are needed to determine whether its clinical benefits outweigh the economic burden and support its broader implementation.

#### 3.1.3. VAC Stent

**Figure 3:**
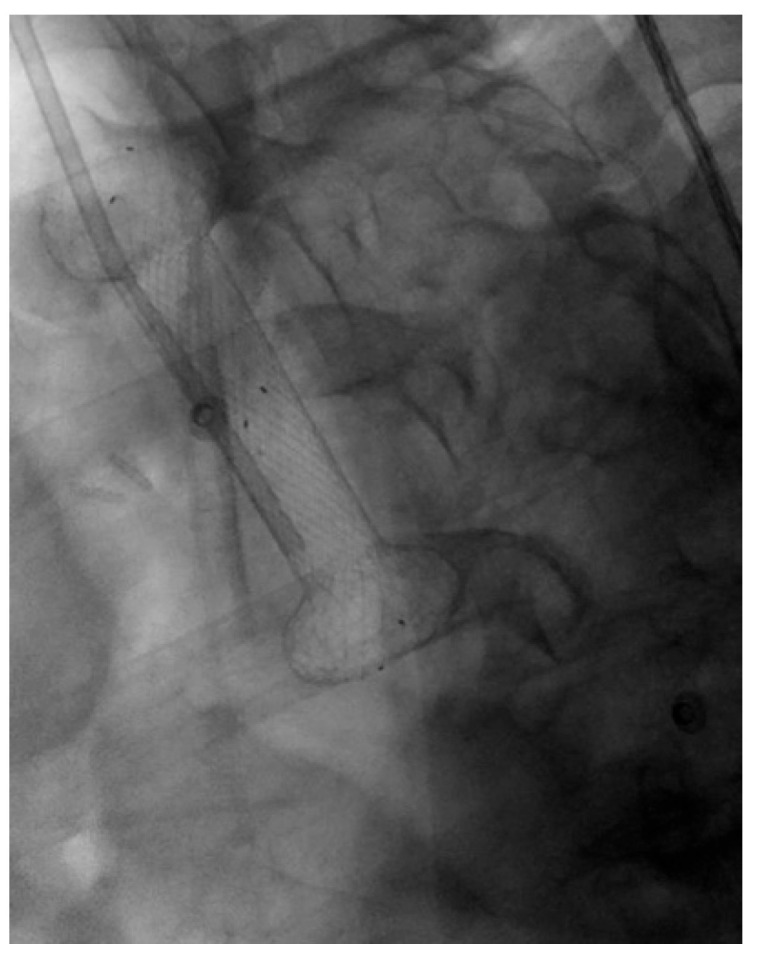
VAC stent for the treatment of an esophagogastric anastomotic fistula following Ivor-Lewis esophagectomy.

An innovative device for the treatment of esophageal AL is the VAC stent, which combines the properties of SEMS and EVT. It consists of a FC-SEMS made of nitinol, coated with a silicone membrane, and externally wrapped with a polyurethane sponge connected to a vacuum pump [16]. The stent is placed endoscopically under radiological guidance and is replaced every 7 days until complete leak healing [16] [Figure 3].

No standardized guidelines exist for setting the suction pressure for the VAC stent. However, the manufacturer recommends an initial pump setting of −125 mmHg for the first 12–24 h, followed by an adjustment to a range of −85 to −100 mmHg. Most existing studies have used continuous suction with negative pressure levels ranging from −65 to −125 mmHg [52,53,54].

The VAC stent has proven effective in the treatment of post-esophagectomy leaks.

Its first documented use dates back to 2020 and involved a patient with an esophago-jejunal AL who achieved complete healing after two VAC stents [55]. Subsequent studies have confirmed its efficacy, even in cases refractory to other endoscopic treatments.

In a study by Chon et al. involving 10 patients, VAC stents were effective in 70% of cases, with higher success when used as a first-line treatment (80%) compared to rescue therapy (60%) [56].

Similarly, a prospective trial enrolling 20 patients reported an overall success rate of 60%, increasing to 71% when VAC stents were used as the initial treatment [57]. In a case series of 10 patients with ALs treated with VAC stents, CS was obtained in all cases [54].

In a multicenter study by Lange J. et al. involving 15 patients, an 80% success rate was reported, with an average of 2.7 VAC stents used per patient [52]. In a study by Bludau et al., 77 patients with upper GI defects (6 spontaneous perforations, 12 iatrogenic perforations, 59 ALs) were treated with a VAC stent, achieving complete esophageal closure in 60 cases (78%) [58].

The VAC stent has also been tested as a preemptive therapy in patients with high-risk anastomoses after neoadjuvant therapy undergoing esophagectomy. In this context, Lange et al. reported complete prevention of ALs in all 10 patients included in their study [53].

Esophageal perforations, including iatrogenic and spontaneous cases (Boerhaave’s Syndrome), represent another established indication [59]. The VAC stent has also shown successful outcomes in managing leaks following bariatric surgery, such as sleeve gastrectomy and Roux-en-Y gastric bypass [60].

VAC stent placement is generally well-tolerated, with a low incidence of AEs. Reported issues include minor mucosal bleeding, stent migration (7%), and esophageal tissue ingrowth [52]. In a study of ten patients, Pattynama et al. reported only one case of anastomotic stricture, which was successfully managed with endoscopic dilation [54]. Notably, no AEs were observed in prophylactic applications.

##### SEMS vs. VAC Stent

Despite the lack of direct comparative studies, the VAC stent appears to be a potentially superior alternative to SEMS.

Its main advantages lie in its integration with vacuum therapy, which enhances the drainage and aspiration of fluid collections associated with ALs, and in its significantly lower migration rate compared to conventional SEMSs. This is attributable to three key features: adhesion to the esophageal wall via negative pressure, the stabilizing effect of the anchored drainage tube, and the dumbbell-shaped flare ends that improve anchorage [16].

The VAC stent is also less prone to stent leaks and related AEs due to its design and suction mechanism [57].

However, it requires constant monitoring and connection to an external vacuum pump, which complicates discharge planning and increases hospital resource use. It must also be replaced more frequently, leading to greater procedural burden, patient discomfort, and costs compared to SEMS. A further limitation is its availability in a single size, whereas SEMSs come in various lengths—a critical factor for tailoring treatment to the location and size of the leak [59].

Future comparative studies are needed to confirm whether the clinical benefits of VAC stent therapy—such as improved outcomes, shorter hospital stays, and fewer interventions—can translate into overall costs that are comparable to, or even lower than, those of current standard treatments [Table 1].

### 3.2. Malignant Dysphagia (Esophageal Cancer)

Esophageal cancer is the eighth most common malignancy worldwide, with over 50% of patients presenting at an advanced stage [65]. Dysphagia, a major complication of advanced disease, significantly impairs both quality of life and nutritional status, posing considerable challenges to subsequent treatment strategies.

#### 3.2.1. Role of Stent

##### Indications and Mechanism of Action

Esophageal stents have been used since 1959, initially inserted via laparotomy to palliate dysphagia caused by esophageal carcinoma [66]. In 1977, an endoscopic technique for stent placement was introduced [67], and by the 1980s, SEMS became widely adopted [68].

According to ESGE guidelines, PC-SEMS or FC-SEMS are the preferred options for the palliative management of malignant dysphagia, offering advantages over laser therapy, photodynamic therapy, and esophageal bypass. Additionally, SEMS placement is indicated for sealing malignant tracheoesophageal or bronchoesophageal fistulas [1].

SEMSs are generally reserved for inoperable patients with a life expectancy of less than three months and poor performance status [1]. For patients expected to survive longer, SEMS may be used in combination with brachytherapy [69]. However, their use as a bridge to surgery or before preoperative chemoradiotherapy is contraindicated [1].

**Figure 4:**
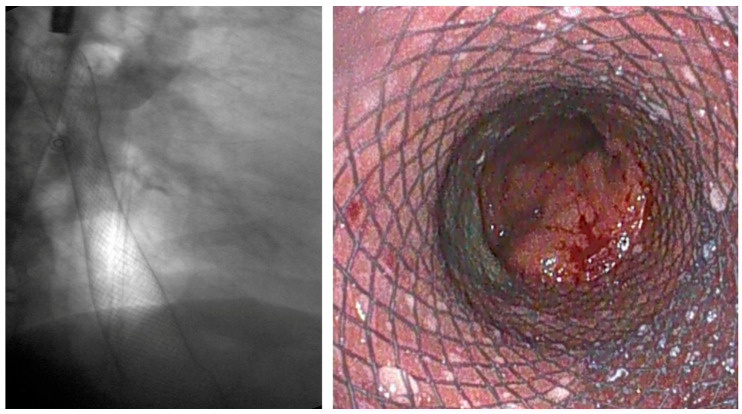
Partially covered esophageal stent (Evolution ^®^, Esophageal Controlled-Release Stent, 25 mm × 12.5 cm, Cook Medical LLC, Bloomington, IN, USA) for obstructing esophageal neoplasia.

Esophageal stent placement typically allows oral intake within 1 to 2 days and reduces dysphagia severity, thereby significantly improving quality of life [Figure 4] [70].

##### Efficacy and Adverse Events

Studies suggest that FC- and PC-SEMS are more effective than UC-SEMS for dysphagia palliation, primarily due to a reduced risk of tumor ingrowth—a major cause of recurrent dysphagia [71,72].

In a multicenter study by Vakil et al., 62 patients with inoperable malignant esophageal obstruction were randomized to receive either FC-SEMS (*n* = 32) or UC-SEMS (*n* = 30) [71]. Both groups showed similar dysphagia improvement after one week, but tumor ingrowth was significantly more frequent in the UC-SEMS group (30% vs. 3%, *p* = 0.005) [71].

A larger study comparing FC-SEMS (esophageal Nitinol Ultraflex stent, Boston Scientific) and UC-SEMS (Nitinol esophageal Strecker stent, Boston Scientific) in 152 patients with malignant esophageal or cardia stenosis confirmed these findings [72]. Restenosis and recurrent dysphagia were significantly more common with UC-SEMS (37% vs. 8%, *p* < 0.0001), and at follow-up, 88% of patients treated with FC-SEMS remained symptom-free, compared to 54% of those treated with UC-SEMS (*p* < 0.0001) [72].

The most frequent AE of SEMS placement for malignant dysphagia is stent migration, which occurs in 4–36% of cases with FC-SEMS [73]. For this reason, PC-SEMSs are more commonly used in clinical practice. In the case of FC-SEMS, fixation techniques such as clips or sutures have been suggested to reduce the risk of migration [74].

Other complications include chest pain and stent obstruction due to tumor ingrowth or overgrowth, both of which can result in recurrent dysphagia [75].

Tumor ingrowth is primarily associated with UC-SEMS, due to their uncovered mesh structure, but may also occur with FC-SEMS in cases of incomplete coverage or prolonged dwell time. Management options include mechanical removal or thermal ablation techniques to restore luminal patency, and in selected cases, the insertion of a second stent may be required.

Tumor overgrowth at the proximal or distal ends of the stent occurs in 4–18% of cases, regardless of stent type [76]. Proper stent sizing—ensuring at least a 2 cm extension beyond the tumor margins—is essential to minimize this risk. When overgrowth occurs, similar endoscopic strategies can be applied, tailored to the location and extent of the obstruction [76].

### 3.3. Benign Strictures

Benign esophageal strictures are a significant clinical concern, with dysphagia being the most common symptom [77]. This typically arises when luminal obstruction exceeds 50% [77]. If left untreated, strictures can lead to malnutrition, aspiration, and a marked decline in quality of life [78]. The etiologies are diverse and include inflammatory conditions (e.g., peptic strictures, eosinophilic esophagitis, Crohn’s disease) as well as iatrogenic causes (e.g., post-radiotherapy, post-endoscopic submucosal dissection/endoscopic mucosal resection/radiofrequency, anastomotic strictures, or caustic ingestion) [79].

#### 3.3.1. Role of Stent

##### Indication and Mechanism of Action

Endoscopic dilation is the first-line treatment for benign strictures. However, in refractory strictures—defined as failure to reach 14 mm after biweekly dilations for more than 5 weeks, or inability to maintain the target diameter within 4 weeks post-dilation—or in complex strictures (length > 2 cm, diameter > 11 mm, irregular or angulated edges), stents are considered a second-line approach [1].

**Figure 5:**
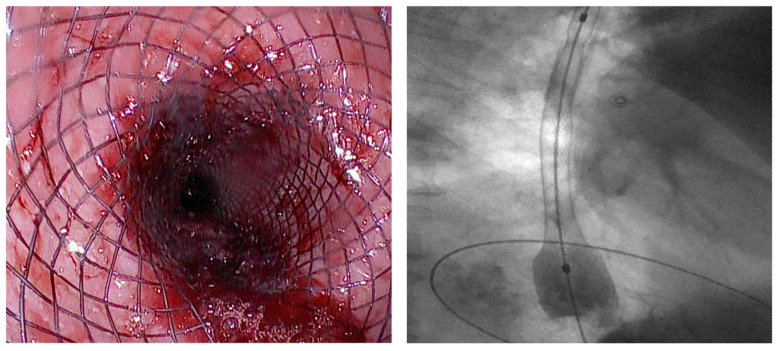
Fully covered SEMS to treat a circumferential stenosis following endoscopic submucosal dissection of an early esophageal squamocellular carcinoma.

Among available options, FC-SEMSs are the most commonly used [1]. These stents are typically left in place for 6 to 12 weeks to allow for stricture remodeling while minimizing complications such as hyperplastic tissue reaction [80]. BDS also represent a valid alternative, particularly in selected cases, as they naturally degrade over time and do not require endoscopic retrieval [Figure 5].

##### Efficacy and Adverse Events

Despite promising short-term symptom relief, CS rates for FC-SEMS in benign esophageal strictures remain modest.

In the study by Kim et al., 55 patients with refractory stenosis treated with FC-SEMS showed a significant reduction in mean dysphagia score from 2.8 to 1.3 (*p* < 0.001). However, during a mean follow-up of 38 months, stricture recurrence requiring balloon dilation occurred in 38 of 55 patients (69%). The patency rates after temporary stenting at 1, 3, and 6 months, and at 1, 2, and 4 years, were 58%, 43%, 38%, 33%, 26%, and 21%, respectively [81].

Liu et al. evaluated the safety and efficacy of FC-SEMS in 24 patients with refractory benign esophagogastric anastomotic strictures, all of whom had previously undergone ≥5 sessions of Savary–Gilliard bougie dilation without meaningful clinical improvement. All patients experienced significant dysphagia relief during and shortly after stent placement, with dysphagia scores decreasing from 3–4 to 0–1. However, at 12-month follow-up, 18 patients (75%) remained symptom-free, while 6 (25%) continued to experience dysphagia [82].

Stent migration is the most frequently reported complication, occurring in 25–35% of cases [81,82]. Liu et al. noted significant acid reflux during stent treatment in 5 of 24 patients, which was managed with medication. Post-procedural chest pain was reported by 21 patients, resolving within six days in all cases [82].

#### 3.3.2. Biodegradable Stents

BDSs have emerged in recent years as a promising alternative for the treatment of benign esophageal strictures [83].

Among the available evidence, Tomonori et al. evaluated the performance of BDS in a cohort of 30 patients with benign strictures, all of whom had a dysphagia score ≤2 and a history of at least five dilation sessions. Fourteen patients (46.7%; 95% CI: 28.3–65.7) experienced sustained symptom improvement up to three months post-placement, with a median dysphagia-free survival of 98 days (95% CI: 68–123). Most AEs—including esophageal/oropharyngeal pain, gastroesophageal reflux, and mucosal hyperplasia—were managed conservatively. However, one patient with a post-chemoradiotherapy (CRT) stricture developed a fatal left atrio-esophageal fistula approximately four months after stent placement [84].

A notable recent advancement is the development of a biodegradable PTX-PLGA-coated magnesium stent. This device incorporates a paclitaxel-loaded polymer and provides a radial force of 83 Newtons, aimed at promoting fibroblast apoptosis and minimizing fibrotic response. In preclinical animal models, the stent maintained esophageal patency for at least three weeks and significantly reduced inflammatory infiltration and fibrous tissue formation [85].

These findings support the potential of BDS as an alternative to removable stents, particularly in patients at higher risk for re-intervention. However, clinical data remains limited, and larger prospective studies are needed to better define their long-term safety and effectiveness.

##### SEMS vs. BDS

Compared to SEMS, BDSs demonstrate comparable efficacy and safety in the management of refractory benign esophageal strictures [86,87,88]. Therefore, ESGE guidelines do not recommend a specific type of stent (FC-SEMS or BDS) for refractory benign esophageal strictures, as no single option has demonstrated clear superiority.

A systematic review and meta-analysis by Fuccio et al., including 18 studies and 444 patients, reported a pooled CS rate of 40.5% for stent placement in refractory benign esophageal strictures, with no significant difference between SEMS (40.1%) and BDS (32.9%). AE rates were also similar, although stent migration was more frequent with SEMS (31.5%) than with BDS (15.3%) [87].

These findings suggest that although BDSs eliminate the need for retrieval and offer a favorable degradation profile, their efficacy and safety are broadly comparable to SEMS. Given their higher upfront cost, careful patient selection based on factors like prior radiotherapy and stricture characteristics remains essential.

#### 3.3.3. LAMS

LAMSs have recently gained attention as a potential option for the treatment of benign esophageal strictures, particularly in refractory cases. Unlike traditional approaches that rely on EUS guidance, LAMSs can be deployed endoscopically under direct visualization, enabling precise positioning across the stricture. Ideally, the catheter is preloaded onto a guidewire previously passed through the stenotic segment. Available in multiple diameters (10 mm, 15 mm, and 20 mm), LAMSs offer flexibility in tailoring the treatment to individual anatomical and clinical needs [12].

A meta-analysis by Giri et al. evaluated the outcomes of LAMS in patients with benign gastrointestinal strictures, including 18 studies and 527 patients [89]. The analysis reported a pooled TS of 99.9% (95% CI: 99.1–100.0; I^2^ = 0.0%). CS was achieved in 93.9% of cases in the short term (95% CI: 90.7–100.0; I^2^ = 39.0%) and in 72.8% in the long term (95% CI: 55.7–90.0; I^2^ = 94.4%) [89]. AEs occurred in approximately 13.5% of cases (95% CI: 8.6–18.5%) and were classified as either periprocedural or delayed. Periprocedural events included bleeding (2.3%), pain (5.7%), and rare cases of perforation (0.1%). Delayed adverse events comprised stent occlusion (1.8%), restenosis (4%), ulceration (0.5%), and migration, the most common, with a rate of 10.6% [89]

**Figure 6:**
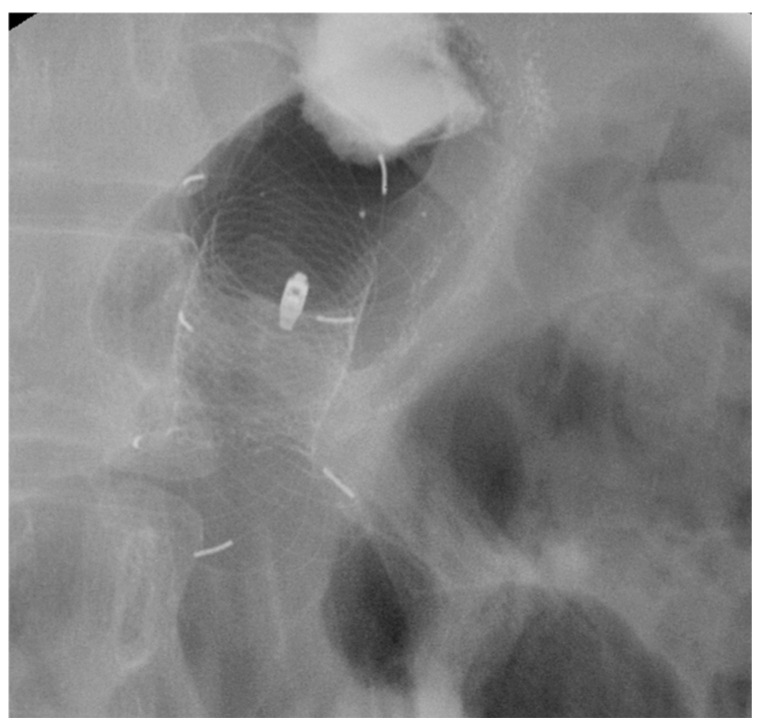
Radiologic view of a LAMS (Nagi^®^ Lumen-Apposing Metal Stent, 16 × 30 mm, Taewoong Medical Co., Ltd., Gwangju, Republic of Korea) placed to treat gastrojejunal anastomotic stricture in a patient who underwent Roux-en-Y gastric bypass.

These results support the use of LAMS as a feasible and effective alternative in selected patients with benign, treatment-refractory esophageal strictures [Figure 6].

##### SEMS vs. LAMS

Current evidence suggests that LAMS may offer improved outcomes compared to SEMS and BDS in the management of benign esophageal strictures.

In the systematic review by Mohan et al. comparing LAMS, FCSEMS, and BDS for the treatment of benign GI strictures (74% of patients had refractory benign esophageal strictures), the pooled CS rate for LAMS was 78.8% (95% CI: 65.8–87.8%; I^2^ = 69.6), which was significantly higher than that reported for FCSEMS (48%) and BDS (34.9%) (LAMS vs. FCSEMS, *p* = 0.001; LAMS vs. BDS, *p* = 0.001) [90]. Regarding safety, LAMSs were associated with a pooled migration rate of 13.7%, which was significantly lower than that of FCSEMS (31.5%; *p* = 0.001) and comparable to BDS (11.5%; *p* = 0.5) [90].

Taken together, these findings suggest that LAMS may represent a more effective and better-tolerated alternative to conventional stents for patients with refractory benign strictures, particularly those that are short in length. Nonetheless, further prospective studies are needed to confirm their long-term safety and to better define their optimal indications.

#### 3.3.4. Incisional Therapy

##### Indication and Mechanism of Action

Incisional therapy is an endoscopic technique primarily used for managing short (<2 cm), fibrotic, benign esophageal strictures—particularly anastomotic strictures and Schatzki rings [91]. Evidence for its use in other stricture types (e.g., corrosive, radiation-induced, peptic, Crohn’s-related) is more limited [92,93].

The procedure involves longitudinal incisions made along the stricture using various endoscopic knives (needle knife, hook knife, or IT-knife), typically parallel to the esophageal axis [94]. Three main techniques are described:

1. Radial Incision (RI): 4–8 radial cuts without removal of scar tissue.
2. Radial Incision and Cutting (RIC): combines incisions with excision of fibrotic tissue.
3. Radial Incision and Selective Cutting (RISC): targets only selected fibrotic segments to minimize the risk of restenosis [95].

**Figure 7:**
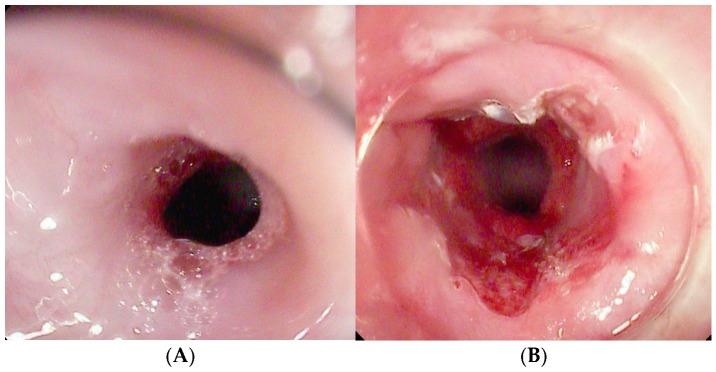
Anastomotic stricture following McKeown esophagectomy (**A**) treated with endoscopic incision (**B**).

A transparent cap may be added to the endoscope tip to improve visualization [Figure 7] [94].

##### Efficacy and Adverse Events

Data increasingly supports the use of incision therapy for the treatment of benign esophageal strictures.

Lee et al. performed endoscopic incision therapy in 24 patients with benign anastomotic strictures following esophagojejunostomy, none of whom had previously undergone dilation. After a two-year follow-up, over 85% of patients remained free from dysphagia after a single treatment session [93].

Excellent outcomes have also been reported for treatment-naïve strictures. A randomized controlled trial by Hordijk et al. compared incision therapy with Savary dilation (Savary–Gilliard bougies, Wilson-Cook Medical Inc., Winston-Salem, NC, USA) in 62 patients with untreated esophageal anastomotic strictures. The study found no significant differences in the mean number of dilations required (2.9; 95% CI, 2.7–4.1 vs. 3.3; 95% CI, 2.3–3.6; *p* = 0.46) or in clinical success rates (80.6% vs. 67.7%, *p* = 0.26; and 96.2% vs. 80.8%, *p* = 0.19) between the two groups [96].

A recent systematic review and meta-analysis by Jimoh et al., including five studies comparing incision therapy with balloon dilation, showed that incision therapy was associated with a significantly lower likelihood of stricture recurrence (OR 0.35, 95% CI 0.13–0.92, *p* = 0.03; I^2^ = 71%). The benefit was even more pronounced in treatment-naïve strictures (OR 0.32, 95% CI 0.17–0.59, *p* = 0.0003; I^2^ = 0%) [97].

AEs associated with incision therapy are generally mild and include bleeding, perforation, and post-procedural pain. The procedure is considered safe when performed by experienced endoscopists. However, caution is advised in cases of long, fibrotic, or highly tortuous strictures, as these may not only be less responsive to treatment but also carry a higher risk of complications [94]. In the meta-analysis by Jimoh et al., three studies found no statistically significant difference in complication rates between incision therapy and balloon dilatation. However, one study reported a significantly higher incidence of perforation in patients treated with balloon dilation, while another found a higher perforation rate with incision therapy [97].

Some authors have described the use of incision therapy as an adjunctive treatment preceding balloon dilation. The rationale lies in the ability of radial incision therapy to weaken the fibrotic tissue and facilitate the subsequent effect of balloon dilation, thereby enhancing disruption of the stricture [98]. Hagiwara et al. evaluated six patients who had failed either balloon dilation or incision therapy alone and were subsequently treated with a combined approach consisting of radial electroincisions followed by balloon dilation. All patients demonstrated objective improvement in symptoms, and no AEs were reported [98].

Compared to traditional stenting, incision therapy offers several advantages. It avoids the placement of foreign bodies, thereby reducing complications such as stent migration, mucosal injury, and the need for repeat procedures for stent removal [94]. Nonetheless, further large-scale studies are warranted to establish the role of incision therapy as a first-line treatment for benign esophageal strictures and to clarify its comparative effectiveness against stent-based strategies [Table 2].

### 3.4. Esophageal Acute Variceal Bleeding

Acute bleeding from esophageal varices accounts for up to 70% of upper gastrointestinal hemorrhage cases in patients with cirrhosis [99]. After initial resuscitation, standard management includes the administration of vasoactive agents (e.g., octreotide, somatostatin, terlipressin) and urgent endoscopic therapy—preferably within 12 h—such as band ligation or sclerotherapy [100].

#### 3.4.1. Role of Stent

##### Indication and Mechanism of Action

In refractory cases—where pharmacological, endoscopic, and radiologic interventions fail—the placement of fully covered self-expandable metal stents (FC-SEMS) is recommended, as outlined in ESGE guidelines [1].

The most studied device is the SX-ELLA DANIS stent, a removable nitinol stent with atraumatic ends, specifically designed for endoscopic deployment and intended for up to 30 days of in situ use. It serves as a temporary measure and a bridge to definitive therapies such as transjugular intrahepatic portosystemic shunt (TIPS) or liver transplantation [101].

##### Efficacy and Adverse Events

FC-SEMSs have shown high efficacy in controlling variceal bleeding, particularly in refractory cases.

A systematic review and meta-analysis by Shao et al., including five studies, reported a TS rate of 96.7% and an initial hemostasis rate of 93.9%, with rebleeding occurring in 13.2% of cases [102]. Similarly, McCarty et al., in a meta-analysis that included 12 studies utilizing specialized SEMSs and two additional stent types, found effective bleeding control in 96% of cases [103].

The most recent meta-analysis reported an immediate bleeding control rate of 91% (95% CI: 82–95%; I^2^ = 0) and a pooled rebleeding rate of 17% (95% CI: 8–32%; I^2^ = 69) [104].

No severe AEs have been reported. The most common complication, as seen in other contexts, is stent migration. Songtanin et al. reported a migration rate of 18% (95% CI: 9–32%; I^2^ = 38) and a pooled ulceration rate of 7% (95% CI: 3–13%; I^2^ = 0) [104].

Compared to balloon tamponade (BT) with the Sengstaken–Blakemore tube, esophageal stents are better tolerated, allow oral intake, and are associated with lower complication rates in the management of refractory acute variceal bleeding [105]. A systematic review and meta-analysis by Rodrigues et al. evaluated both BT and esophageal stenting for bleeding control, reporting a pooled short-term failure rate of 35.5% for BT compared to 12.7% for esophageal stenting. The rate of AEs was similar between the two modalities, occurring in over 20% of cases [106].

## 4. Gastroduodenal Tract—Gastric Outlet Obstruction

Gastric outlet obstruction (GOO) is defined as a mechanical or functional blockage of the distal stomach, pyloric canal, or proximal duodenum, resulting in impaired gastric emptying and disruption of the normal passage of gastric contents into the small intestine. It may arise from intrinsic luminal narrowing, extrinsic compression, or motility disorders such as gastroparesis [107,108,109,110]. Among these, mechanical causes are far more prevalent, with malignancies accounting for approximately 50–80% of all cases [111,112]. Clinically, GOO presents with a constellation of symptoms including epigastric pain, postprandial nausea and vomiting, early satiety, weight loss, and progressive malnutrition. These manifestations significantly impair quality of life and often necessitate prompt palliation.

### 4.1. Malignant GOO

The two most frequent malignancies associated with GOO are distal gastric carcinoma and pancreatic adenocarcinoma [113]. Less common causes include cholangiocarcinoma, ampullary or duodenal adenocarcinoma, and lymphoma [114].

To guide therapeutic decisions, malignant GOO can be anatomically classified into three types [115]:

- Type I: Stenosis at the duodenal bulb without papillary involvement.
- Type II: Obstruction in the second part of the duodenum involving the papilla—requiring combined palliation of both gastric obstruction and biliary drainage.
- Type III: Obstruction in the third portion of the duodenum, sparing the papilla [115].

#### 4.1.1. Role of Stent

##### Indications and Mechanism of Action

For years, enteral SEMSs were considered the main minimally invasive option for the management of malignant GOO. They served as an alternative to surgical gastrojejunostomy (S-GE), offering reduced perioperative risk and faster recovery compared to surgery [116,117]. However, due to their limited long-term patency and higher rates of stent-related complications, SEMS were generally reserved for patients with poor performance status and limited life expectancy, whereas S-GE was preferred in those with better overall condition and longer anticipated survival [116,117].

**Figure 8:**
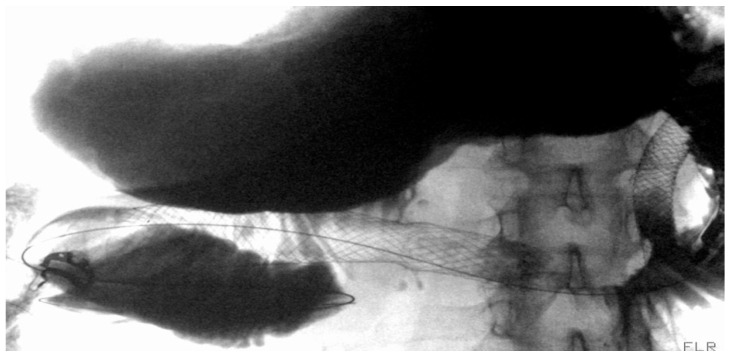
Patient with pancreatic carcinoma causing duodenal stenosis distal to the papillary region, with a biliary stent in place. A 9 cm TTS evolution metal stent was initially deployed but subsequently migrated distally. A second 9 cm TTS WallFlex metal stent was then placed within the first and deployed into the duodenal bulb.

Stent selection between FC and UC-SEMS was traditionally made on a case-by-case basis. In patients with GOO due to biliopancreatic malignancies, biliary drainage was typically performed first, as early placement of a duodenal SEMS could hinder subsequent ERCP and biliary access [118,119]. UC-SEMSs were generally preferred when future access to the papilla was anticipated, whereas FC-SEMSs were thought to interfere with papillary cannulation and thus complicate further management [Figure 8] [118,119].

##### Efficacy and Adverse Events

SEMS placement has traditionally been effective in rapidly relieving symptoms of malignant GOO; however, its long-term durability remains a key limitation [120,121].

A comprehensive meta-analysis by Mintziras et al. (2019), which included 1306 patients treated with SEMS, reported high TS rates (83.3–100%) and CS rates ranging from 75% to 100% [122]. Despite these favorable short-term outcomes, tumor ingrowth, overgrowth, and stent migration often resulted in recurrent obstruction requiring reintervention. The most common causes were stent migration or dislocation (34.2%), bowel perforation (12.5%), and occlusion (14.2%) [122]. These complications often necessitated additional endoscopic procedures, contributing to increased hospital readmissions and adversely affecting patient quality of life.

In terms of stent selection, FC-SEMSs were often favored for their enhanced patency, despite a higher migration risk (16–25%), while UC-SEMS provided better anchorage but were more susceptible to tissue ingrowth (4–26%) [123,124].

Although SEMSs offered a minimally invasive approach for malignant GOO palliation, their long-term effectiveness was limited by device-related dysfunctions. As a result, SEMSs have increasingly been replaced by EUS-GE with LAMS, a technique that provides more durable symptom relief and, in selected patients, has shown superior clinical outcomes even when compared to S-GE.

#### 4.1.2. LAMS

##### Indication and Mechanism of Action

EUS-guided gastroenterostomy (EUS-GE) is an advanced endoscopic technique that creates a gastrojejunal anastomosis using a LAMS under EUS guidance [125].

The technique was developed to combine the minimally invasive nature and rapid recovery associated with enteral SEMS with the long-term efficacy of surgical gastrojejunostomy (S-GE). Unlike SEMSs, which are placed across the malignant stricture and often suffer from tumor ingrowth or migration, EUS-GE creates a bypass distant from the obstruction, potentially reducing the risk of stent dysfunction and improving durability.

The procedure involves deploying a LAMS between the stomach and a jejunal loop distal to the obstruction. Loop identification can be achieved through the following:

- Direct technique: puncture of the jejunal loop with a 19G needle and contrast injection to confirm position [126].
- Device-assisted EUS-GE: balloon or enteroscope passed across the stenosis to aid EUS visualization and targeting [127].
- Wireless Endoscopic Simplified Technique (WEST): described by Bronswijk et al. in 2020 and currently the most widely used technique [128], this approach involves jejunal distension via a nasoenteric tube with saline and dye, followed by “free-hand” single-step LAMS deployment under EUS guidance [128].

**Figure 9:**
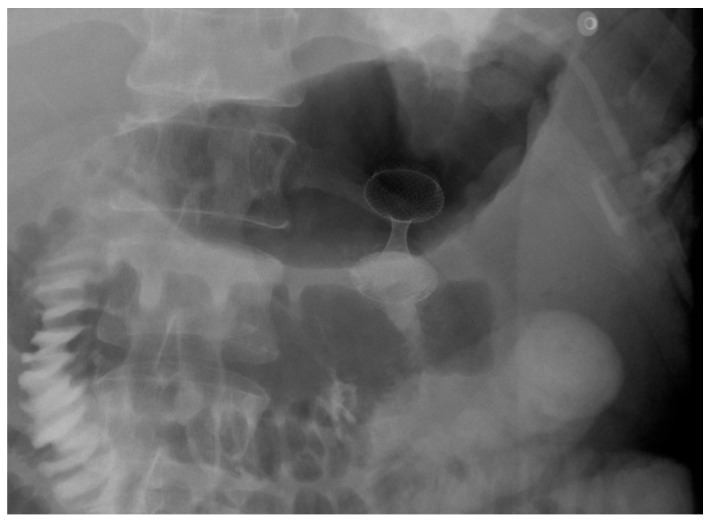
Radiologic view of a LAMS (Axios™, Boston Scientific, Marlborough, MA, USA) placed using EUS-guided technique to palliate a GOO in a patient with pancreatic adenocarcinoma.

According to ESGE guidelines, the primary indication for EUS-GE is malignant GOO, as an alternative to SEMS or S-GE [3]. The procedure should be reserved for expert operators and performed in tertiary referral centers, following multidisciplinary discussion [3]. With improved survival due to neoadjuvant therapies—particularly in pancreatic adenocarcinoma—EUS-GE is increasingly used not only as palliation but also as a bridge-to-surgery in selected patients, with growing supporting evidence [Figure 9] [129].

##### Efficacy and Adverse Events

Building on its technical rationale and increasing adoption in expert centers, EUS-GE has demonstrated high efficacy and a favorable safety profile in clinical practice.

In the prospective cohort study by Vanella et al., which included 70 patients with malignant GOO (75.7% with pancreatic cancer, 60% with metastatic disease), both TS and clinical success CS rates were 97.1% [130]. After a median follow-up of 105 days (IQR 49–187), 61.4% of patients were able to resume chemotherapy, with a median time-to-chemotherapy of 19 days (IQR 14–26). Symptom recurrence occurred in 7.6% of cases, with a median time to recurrence of 78 days (IQR 28–164). Kaplan–Meier analysis estimated a mean symptom-free survival of 480 days (95% CI 426–534), with probabilities of 96.7%, 90.6%, and 85% at 3, 6, and 12 months, respectively [130].

Similarly, a study by Trieu et al. evaluating 207 patients undergoing EUS-GE reported sustained long-term efficacy (CS 97.2%; TS 95.7%) with LAMS remaining in place in 63.6% of patients for over 3 months, and in 21% for more than one year. The overall rate of late AEs was 3.4%, confirming the favorable safety profile of the procedure [131].

Early AEs, primarily EUS-GE-related, include misdeployment, perforation, non-perforative peritonitis, and bleeding [132]. Late AEs consist mainly of delayed stent migration and LAMS occlusion [132]. A meta-analysis of 36 studies evaluating EUS-GE-associated AEs reported an overall complication rate of 13.0%. Interestingly, significantly fewer events were observed when using WEST (8.4%) compared to other methods (17.3%) [132]. Serious AEs were rare (1.2%), and procedure-related mortality was extremely low (0.3%). Misdeployment was the most frequent complication (4.6%), and in this case, it occurred less frequently with the free-hand technique (2.8%) than with mixed approaches (7.1%) [132].

A retrospective multicenter study by Ghandour et al. further classified LAMS misdeployment into four types [133]:

- Type 1 (63.1%): distal flange in the peritoneum, proximal in the stomach, without enterotomy—managed with LAMS removal and OTSC placement.
- Type 2 (30.4%): distal flange in the peritoneum, proximal in the stomach, with confirmed enterotomy—managed with repeat LAMS or LAMS-in-LAMS bridging.
- Type 3 (2.2%): distal flange in the small bowel, proximal in the peritoneum—managed surgically.
- Type 4 (4.3%): distal flange in the colon, proximal in the stomach—managed conservatively or surgically after tract maturation.

##### Sems vs. LAMS

Comparative studies evaluating EUS-GE versus duodenal stent placement have shown that EUS-GE offers a higher initial CS rate, a lower risk of stent dysfunction requiring secondary endoscopic intervention, and better long-term symptom control, ultimately improving patients’ quality of life [134,135].

A meta-analysis by Miller et al. (2023), including 16 studies and 1541 patients, confirmed superior CS rates for EUS-GE compared to both SEMS and surgical gastrojejunostomy (S-GE) (OR vs. SEMS or surgery: 2.60, 95% CI 1.58–4.28; OR vs. SEMS alone: 5.08, 95% CI 3.42–7.55) [136,137].

In a matched subgroup analysis within the prospective study by Vanella et al., EUS-GE achieved a CS rate of 100% versus 75.0% with duodenal stenting (*p* = 0.006), and a significantly lower symptom recurrence rate (3.7% vs. 33.3%, *p* = 0.02) [130].

The first RCT to directly compare EUS-GE and SEMS as palliative treatments for malignant GOO was conducted across seven high-volume centers by Teoh et al. The study enrolled 97 patients with GOO due to unresectable gastro-duodenal or pancreatobiliary malignancies, who were randomized to receive either EUS-GE (48 patients) or SEMS placement (49 patients) [138]. EUS-GE demonstrated a significantly lower 6-month reintervention rate compared to SEMS (4% vs. 29%, *p* = 0.0020; RR 0.15, 95% CI 0.04–0.61) and significantly longer stent patency [138].

Regarding safety, a network meta-analysis showed that EUS-GE was associated with a lower risk of AEs compared to SEMS (RR 0.58) and a comparable risk to surgical gastrojejunostomy (RR 1.43) [137]. Among the three modalities, EUS-GE had the highest probability of being the safest treatment option (P score: 0.99), followed by SEMS (P score: 0.62) [137]. These findings were corroborated by another meta-analysis reporting significantly fewer adverse events for EUS-GE compared to SEMS or SGJ combined (OR 0.34; 95% CI 0.20–0.58) [136].

Although LAMS are more expensive than duodenal SEMS, recent evidence shows that EUS-GE is cost-effective compared with SEMS [139]. A recent study by Ramai et al. compared EUS-GE with LAMS to conventional duodenal SEMSs in patients with unresectable or metastatic malignant GOO [139]. Costs were estimated based on Medicare reimbursement rates, and effectiveness was expressed in quality-adjusted life years (QALYs). Duodenal SEMS placement was associated with an average cost of USD 22,748 and 0.31 QALYs, whereas EUS-GE with LAMS resulted in a cost of USD 32,254 and 0.53 QALYs. This translated into an incremental cost of USD 9507 for an additional 0.23 QALYs, yielding an incremental cost-effectiveness ratio (ICER) of USD 41,994/QALY, which is well below the accepted willingness-to-pay threshold of USD 100,000/QALY. In probabilistic sensitivity analyses, EUS-GE was favored in 62% of 10,000 simulations, with model sensitivity driven mainly by differences in mortality between SEMS and EUS-GE [139]. Overall, the study concluded that EUS-GE represents a cost-effective palliative alternative to duodenal stenting in malignant gastric outlet obstruction [139].

This innovative approach represents an excellent option for selected patients. However, it is currently limited to highly specialized centers. Further studies and broader dissemination are essential to establish EUS-GE as a widely accessible and potentially first-line therapeutic strategy for malignant GOO [Table 3].

### 4.2. Benignant GOO

Benignant GOO refers to a non-malignant narrowing of the distal stomach, pylorus, or duodenum, which impedes the normal passage of gastric contents into the small intestine. Before the advent of effective Helicobacter pylori eradication therapy, peptic ulcer disease was the most common cause of benign GOO, accounting for approximately 90% of cases [140]. Today, benign etiologies are more varied and include Crohn’s disease, eosinophilic gastroenteritis, caustic-induced strictures, and extrinsic compressions due to acute pancreatitis or necrotic peripancreatic collections [112].

For benign strictures, endoscopic balloon dilation remains the first-line treatment, especially in partial obstruction [141]. However, its efficacy often requires multiple sessions, and its role is limited in complex or refractory cases.

#### 4.2.1. Role of Stent

FC-SEMSs have been explored as a treatment option in patients refractory to dilation or unfit for surgery. However, their use in benign settings remains controversial. In selected cases—such as peptic ulcer-related strictures unresponsive to dilation—temporary SEMS placement has shown favorable outcomes, with stent removal typically performed after approximately 12 weeks of treatment [142].

Despite these isolated reports, stent migration, tissue ingrowth, and difficulties in removal represent significant limitations of SEMS in the benign setting. Moreover, in cases of extrinsic compression, available evidence suggests that neither dilation nor stenting provides effective long-term relief [143].

#### 4.2.2. LAMS

Emerging data supports the use of EUS-GE in selected patients with benign GOO, particularly those who are ineligible for surgery. The technique has been proposed both as a definitive treatment—such as in chronic pancreatitis or peptic ulcer-related obstruction—and as a bridge to elective surgery [144].

A systematic review and meta-analysis by Canakis et al. included 10 studies and 181 patients with benign GOO unfit for surgery. Reported etiologies included chronic pancreatitis (*n* = 48), acute pancreatitis (*n* = 41), peptic strictures (*n* = 19), and post-surgical anastomotic strictures (*n* = 13). The mean procedure time was 66 min [145]. Pooled TS and CS rates were 95% (95% CI: 87.3–98.2; I^2^ = 0%) and 90.6% (95% CI: 81.1–95.4; I^2^ = 0%), respectively [145]. Pooled TS and CS rates were 95% (95% CI: 87.3–98.2; I^2^ = 0%) and 90.6% (95% CI: 81.1–95.4; I^2^ = 0%), respectively [145]. AEs occurred in 11% of cases (95% CI: 6.1–22.3; I^2^ = 38.7%), while reintervention was required in 7% (95% CI: 2.2–21.0; I^2^ = 35.9%) [145].

Despite these promising results, current ESGE guidelines restrict the use of EUS-GE to malignant GOO, primarily due to concerns regarding stent misdeployment [3]. However, a study by Gonzalez et al. directly compared the Direct EUS-GJ technique with WEST in benign indications. The misdeployment rate was significantly lower with WEST (3.7%) compared to the Direct technique (18%, *p* < 0.05). Notably, the study showed comparable technical and clinical success rates in benign and malignant GOO, with overall misdeployment rates remaining below 4% when using WEST [146].

## 5. Conclusions and Future Perspectives

The landscape of endoluminal stenting in 2025 underscores both its benefits and emerging limitations in managing GI diseases.

Stents remain effective, cost-efficient, and minimally invasive for certain conditions. However, their role is increasingly challenged in other scenarios where alternative devices and procedures have surpassed stents in terms of efficacy, cost-effectiveness, and lower rates of AEs.

Equally relevant is the consideration of training requirements and learning curves, especially for technically demanding procedures such as EUS-guided stent placement. These techniques demand not only advanced endoscopic skills but also prolonged mentoring, simulation-based practice, and high procedural volumes to achieve proficiency. At present, access to such interventions is largely restricted to tertiary referral centers, which raises concerns about equity of care and the scalability of these innovations.

In the coming years, stents and these alternative therapies will likely coexist—either complementing each other or competing for dominance, depending on the clinical context. Future randomized controlled trials are essential to compare these approaches directly and establish their relative efficacy, safety, and long-term outcomes. Such research will be critical to fostering a more personalized approach to treatment, where the choice between stent and alternative therapy is tailored to patient characteristics, pathology, and procedural complexity. In parallel, innovation in stent technology—such as enhanced anti-migration designs, bioresorbable materials with tunable degradation rates, and anti-biofilm coatings—will further expand their utility. Altogether, these advancements point toward an increasingly sophisticated and patient-centered framework for endoscopic management of upper gastrointestinal diseases.

## Figures and Tables

**Table 1:** Endoscopic treatment of esophageal leaks: CS and AEs.

Authors	Study Design	N° Patients	Indications	Clinical Success %	Adverse Events %
SEMS					
Anderloni et al. [19]	Retrospective	49	AL post esophageal surgery	60.5	38
Plum et al. [20]	Retrospective	70	AL post esophageal surgery	70	28.6
Segura et al. [21]	Retrospective	20 3	AL post esophageal surgery Esophageal perforations	75	21.7
Fischer et al. [61]	Retrospective	11	AL post esophageal surgery	100	0
Iglesias Jorquera et al. [23]	Retrospective	25	AL post esophageal surgery	84	28
Mennigen et al. [62]	Retrospective	45	AL post esophageal surgery	63.3	36.7
Mandarino et al. [17]	Retrospective	37	AL post esophageal surgery	62.2	22.7
Licht et al. [63]	Retrospective	49	AL post esophageal surgery	88	3.2
Schweigert et al. [64]	Retrospective	25	AL post esophageal surgery	76.5	23.5
EVT					
Richter et al. [41]	Prospective	69 33	AL post esophageal surgery Esophageal perforations	91 76	NA
Jung et al. [38]	Retrospective	119	AL post esophageal surgery Esophageal perforations	70.6	10.9
Momblan et al. [39]	Prospective	89 13	AL post esophageal surgery Esophageal perforations	82	5.9
Luttikhold et al. [40]	Retrospective	27	AL post esophageal surgery Esophageal perforations	89	7
VAC STENT					
Chon et al. [56]	Prospective	5 5	AL post esophageal surgery Esophageal perforations	70	0
Chon et al. [57]	Prospective	18 2	AL post esophageal surgery Esophageal perforations	60–71	0
Pattynama et al. [54]	Case series	8 2	AL post esophageal surgery Esophageal perforations	100	0
Lange et al. [52]	Prospective	11 4	AL post esophageal surgery Esophageal perforations	80	7
Blundau et al. [58]	Retrospective	59 18	AL post esophageal surgery Esophageal perforations	78	NA

AL: anastomotic leak; NA: not applicable; SEMS: self-expanding metal stent; EVT: endoscopic vacuum therapy.

**Table 2:** Endoscopic treatment of benign esophageal strictures: CS and AEs.

Authors	Study Design	N° Patients	Indications	Clinical Success %	Adverse Events %
SEMS					
Kim et al. [81]	Retrospective	55	Benign esophageal strictures	58—1 month 43—3 months 38—6 months 33—1 year 26—2 years 21—4 years	31—tissue hyperproliferation 24—severe pain 25—stent migration
Liu et al. [82]	Prospective	24	Benign anastomotic esophageal strictures	75—1 year	72.4—moderate chest pain 3.4—stent migration 17.2—reflux
Fuccio et al. [87]	Metanalysis	227	Benign esophageal strictures	40.1	21.9
Mohan et al. [90]	Metanalysis	342	Benign GI strictures	48	31.5
BDS					
Tomonori et al. [84]	Non-randomized prospective trial	30	Benign esophageal strictures	46.7	NA
Fuccio et al. [87]	Metanalysis	77	Benign esophageal strictures	32.9	21.9
Mohan et al. [90]	Metanalysis	226	Benign GI strictures	34.9	11.5
LAMS					
Giri et al. [89]	Metanalysis	527	Benign GI strictures	93.9	13.5
Mohan et al. [90]	Metanalysis	192	Benign GI strictures	78.8	29.9
Incisional therapy					
Lee et al. [93]	Prospective	24	Benign anastomotic esophageal strictures	80.6	0
Hordijk et al. [96]	Prospective	31	Benign esophageal anastomotic strictures	78	NA

SEMS: self-expanding metal stent; NA: not applicable; BDS: biodegradable stent; LAMS: lumen apposing metal stent; GI: gastrointestinal.

**Table 3:** Endoscopic treatment of gastric outlet obstruction: CS and AEs.

Authors	Study Design	N° Patients	Indications	Clinical Success %	Adverse Events %
SEMS					
Mintziras et al. [122]	Metanalysis	1306	Malignant gastric outlet obstruction	75–100	34.2—stent migration 12.5—perforation 14.2—stent disfunction
Teoh et al. [138]	RCT	49	Malignant gastric outlet obstruction	92	24
LAMS					
Vanella et al. [130]	Prospective	70	Malignant gastric outlet obstruction	97.1	12.9
Trieu et al. [131]	Retrospective	207	Gastric outlet obstruction	97.2	4.8—Early AEs 3.4—Late AEs
Teoh et al. [138]	RCT	48	Malignant gastric outlet obstruction	100	23

SEMS: self-expanding metal stent; LAMS: lumen-apposing metal stent; AEs: adverse events.

## Data Availability

No new data were created.

## Abbreviations

GI, gastrointestinal; EUS-GE, EUS-guided gastroenterostomy; GOO, gastric outlet obstruction; EVT, endoscopic vacuum therapy; SEMS, self-expanding metal stents; FC-SEMS, fully covered SEMS; PC-SEMS, partially covered SEMS; UC-SEMS, uncovered SEMS; BDS, biodegradable stent; LAMS, lumen-apposing metal stent; AL, anastomotic leaks; CS, clinical success; AE, adverse events; TTSC, Through-The-Scope Clips; OTSC, Over-The-Scope Clips; S-GE, surgical gastrojejunostomy.
